# Guidelines for histopathological specimen examination and diagnostic reporting of primary bone tumours

**DOI:** 10.1186/2045-3329-1-6

**Published:** 2011-07-25

**Authors:** D Charles Mangham, Nicholas A Athanasou

**Affiliations:** 1 Department of Musculoskeletal Pathology, Robert Jones and Agnes Hunt Orthopaedic Hospital, Oswestry SY10 7AG and Department of Musculoskeletal Pathology, Royal Orthopaedic Hospital, Birmingham, B31 2AP, UK; 2 NDORMS, University of Oxford, Department of Pathology, Nuffield Orthopaedic Centre, Oxford, OX3 7LD, UK

## Abstract

This review is intended to provide histopathologists with guidelines for clinical assessment, specimen handling and diagnostic reporting of benign and malignant primary bone tumours. Information from radiology, surgical, oncology and other clinical colleagues involved in the diagnosis and treatment of primary bone tumours should be properly assessed before undertaking a structured approach to specimen handling and histological reporting. This ensures that the information needed for planning appropriate treatment of these complex tumours is provided. Consistency in diagnostic evaluation with respect to both terminology and report content facilitates liaison at multidisciplinary bone tumour meetings and collaboration between cancer units and networks, as well as providing a common database for audit of the clinical, radiological and pathological aspects of bone tumours.

## 1. Introduction

This review is intended to provide histopathologists with guidelines for specimen handling and diagnostic reporting of benign and malignant primary bone tumours; the principles of specimen handling required for assessment of secondary bone tumours are similar. As many primary bone tumours are uncommon or rare, experience in diagnostic orthopaedic pathology is required to maintain a high standard of histological reporting of bone tumours; participation in an external quality assessment (EQA) scheme which includes bone tumour pathology is recommended.

Close cooperation is needed between the histopathologist and radiology, surgical, oncology and other clinical colleagues in the diagnosis and treatment of bone tumours; consensus clinical practice guidelines for managing bone sarcomas have been recently published [1,2]. All primary malignant bone tumour cases should be discussed at a properly constituted sarcoma multidisciplinary team (MDT) meeting.

## 2. Classification, grading & staging of primary bone tumours

Primary benign and malignant bone tumours vary widely in their clinical behaviour and pathological features. The nomenclature and classification of primary bone tumours is based mainly on the pathway of tumour cell differentiation; this is usually evidenced by the type of connective tissue matrix formed by tumour cells. The histogenesis of many primary bone tumours, however, is not known and a number of bone tumours are by convention classified by distinct morphological or clinicopathological features (eg giant cell tumour of bone) or by karyotypic and molecular genetic abnormalities (eg Ewing's sarcoma) [3,4]. The 2002 World Health Organisation (WHO) classification of bone tumours is recommended for histological reporting of bone tumours as it is well-recognised and widely employed internationally [3]. [Table 1].

**Table 1:** WHO classification and SNOMED codes of primary bone tumours [3]

Cartilage tumours		Giant cell tumours	
Osteochondroma	9210/0*	Giant cell tumour	9250/1
Chondroma	9220/0	Malignancy in giant cell tumour	9250/3
Enchondroma	9220/0		
Periosteal chondroma	9221/0		
Multiple chondromatosis	9220/1	**Notochordal tumours**	
Chondroblastoma	9230/0	Chordoma	9370/3
Chondromyxoid fibroma	9241/0		
Chondrosarcoma	9220/3		
Central, 1° and 2°	9220/3	**Vascular tumours**	
Peripheral	9221/3	Haemangioma	9120/0
Dedifferentiated	9243/3	Angiosarcoma	
Mesenchymal	9240/3		
Clear cell	9242/3		
		**Smooth muscle tumours**	
		Leiomyoma	8890/0
**Osteogenic tumours**			
Osteoid osteoma	9191/0		
Osteoblastoma	9200/0		
Osteosarcoma	9180/3	**Lipogenic tumours**	
Conventional	9180/3	Lipoma	8850/0
Chondroblastic	9181/3	Liposarcoma	8850/3
Fibroblastic	9182/3		
Osteoblastic	9180/3		
Telangiectatic	9183/3	**Neural tumours**	
Small cell	9185/3	Neurilemmoma	9560/0
Low grade central	9187/3		
Secondary	9180/3		
Parosteal	9192/3	**Miscellaneous tumours**	
Periosteal	9193/3	Adamantinoma	9261/3
High grade surface	9194/3	Metastatic malignancy	
**Fibrogenic tumours**		**Miscellaneous lesions**	
Desmoplastic fibroma	88230	Aneurysmal bone cyst	33640
Fibrosarcoma	88103	Simple cyst	33400
		Fibrous dysplasia	74910
		Osteofibrous dysplasia	92620
**Fibrohistiocytic tumours**		Langerhans cell histiocytosis	97511
Benign fibrous histiocytoma	8830/0	Erdheim-Chester disease	77920
Malignant fibrous histiocytoma	8830/3	Chest wall hamartoma	75580
**Ewing sarcoma**			
Ewing sarcoma	9260/3		
**Haematopoietic tumours**			
Plasma cell myeloma	9732/3		
Malignant lymphoma, NOS	9590/3		

Histological grading of a bone sarcoma provides a guide as to its biological behaviour and is based largely on the degree of cellular and nuclear pleomorphism, cellularity, mitotic activity and the extent of tumour necrosis [3-7]. Some high-grade monomorphic tumours, (such as Ewing's sarcoma), and some other specific tumour types cannot be graded accurately in this way and the tumour grade is defined by the specific histological type or subtype. A modified version of the grading recommendations of the College of American Pathologists is shown in Table 2 (7). In this scheme, conventional chondrosarcoma is divided into Grade 1 (low), Grade II (intermediate) and Grade III (high) on the basis of cellularity and nuclear pleomorphism, as these features have been shown to correlate with prognosis [7,8]; some specific chondrosarcoma subtypes are considered high-grade (eg mesenchymal chondrosarcoma), or low-grade (clear cell chondrosarcoma) Conventional osteosarcoma and most osteosarcoma subtypes are considered high-grade with the exception of low-grade central osteosarcoma and periosteal osteosarcoma (both low-grade) and periosteal osteosarcoma (intermediate-grade)[7,9]. Most chordomas generally behave as intermediate-grade locally aggressive tumours which frequently recur and can metastasise. Osteofibrous dysplasia-like (differentiated) adamantinoma rarely metastasises and is considered low-grade whereas classic adamantinoma has significant metastatic potential and is considered intermediate-grade. Grading is not useful in predicting the behaviour of conventional giant cell tumour of bone, but malignant giant cell tumour is considered high-grade. Other sarcoma types that develop in both soft tissue and bone are graded according to the French Federation of Cancer Centres Sarcoma Group (FNCLCC) grading system [10].

**Table 2:** Bone sarcoma grading

Grade 1	Low-grade central osteosarcoma
	Parosteal osteosarcoma
	Low-grade chondrosarcoma
	Clear cell chondrosarcoma
	Osteofibrous dysplasia-like adamantinoma
Grade 2	Periosteal osteosarcoma
	Intermediate - grade chondrosarcoma
	Classic adamantinoma
	Chordoma
Grade 3	Osteosarcoma (conventional, telangieclatic, small cell, secondary,
	high-grade surface)
	Ewing's sarcoma
	High-grade chondrosarcoma
	Dedifferentiated chondrosarcoma
	Mesenchymal chondrosarcoma
	Dedifferentiated chordoma
	Malignant giant cell tumour

(Modified from reference [7])

Bone sarcomas are staged using either the American Joint Committee on Cancer (AJCC) TNM staging system or the Musculoskeletal Tumour Society (MSTS) Staging System (SSS) [11-14]. (Tables 3, 4). The AJCC and MSTS systems have many features in common. The most recent version of the AJCC staging of primary bone malignancies has subdivided Stage 1 and Stage II tumours on the basis of tumour size rather than intraosseous or extraosseous extent of the tumour (as in the MSTS system). Specifically, tumour size of less than 8 cm maximum dimension is considered a favourable prognostic indicator. The current AJCC system also recommends that tumours with skip metastases are classified separately as Stage III, and that Stage IV tumours associated with distant metastases are subdivided on the basis of whether these are only to the lung (Stage IVA) or to other sites, including bone (Stage IVB). MSTS staging uses a two-grade system of histological grading whereas AJCC staging uses a 4-grade system, with grade 1 and 2 effectively considered low-grade and grades 3 and 4, high grade [14]. Formal staging of a bone tumour should be carried out at a sarcoma MDT meeting where clinical, radiological and histological information can be obtained.

**Table 3:** TNM staging system for bone tumours

T - Primary tumour				
TX	Primary tumour cannot be assessed			
T0	No evidence of primary tumour			
T1	Tumour 8 cm or less in greatest dimension			
T2	Tumour more than 8 cm in greatest dimension			
T3	Discontinuous tumours in the primary bone site			
**N - Regional lymph nodes**				
NX	Regional lymph nodes cannot be assessed			
N0	No regional lymph node metastasis			
N1	Regional lymph node metastasis			
**M - Distant metastasis**				
MX	Distant metastasis cannot be assessed			
M0	No distant metastasis			
M1	Distant metastasis			
M1a	Lung			
M1b	Other distant sites			
**G - Histologic grade**				
GX	Grade cannot be assessed			
G1	Well differentiated - low grade			
G2	Moderately differentiated - low grade			
G3	Poorly differentiated - high grade			
G4	Undifferentiated - high grade*			
**Stage grouping**				
**Stage**	**Tumour (T)**	**Node (N)**	**Metastasis (M)**	**Grade (G)**
Stage IA	T1	NO	MO	G1, 2 low grade
Stage IB	T2	NO	MO	G1, 2 low grade
Stage IIA	T1	NO	MO	G3, 4 high grade
Stage IIB	T2	N0	MO	G3, 4 high grade
Stage III	T3	NO	MO	Any G
Stage IVA	Any T	NO	M1a	Any G
Stage IVB	Any T	N1	Any M	Any G
	Any T	Any N	M1b	Any G
				

*Ewing's sarcoma is classified as high grade.

**Table 4:** Musculoskeletal Tumour Society staging system for bone tumours

**Benign tumours (G0)**
Stage I - Inactive, latent (G0)
Stage II - Active (G0)
Stage III - Aggressive (G0)
**Malignant tumours**
Stage I - Low grade (G1)
Stage II - High grade (G2)
Stage III - Low or high grade tumours with metastases
**Subdivisions**
A - Intracompartmental
B - Extracompartmental

## 3. Clinical & radiological information required for the pathological diagnosis of bone tumours

Histopathological assessment of a bone tumour needs to take into account the clinical background and features of the lesion, its radiological appearances and the results of relevant laboratory investigations [15,16] (Tables 5, 6). Diagnostic evaluation and treatment should optimally be carried out at a centre which specialises in the diagnosis and treatment of bone tumours.

**Table 5:** BONE TUMOUR DIAGNOSIS: CLINICAL FEATURES

• Age (date of birth) and sex of the patient.
• Racial background [17]
• A record of the anatomical bone involved by tumour.
• Clinical features associated with the tumour, such as nature and duration of signs and symptoms, including the presence or absence of pain, swelling, deformity, and relation to a previous traumatic episode.
• The presence or absence of a pre-existing or concomitant skeletal disease, history of familial syndrome or other relevant disease predisposing to tumour development.
• Occupational or treatment (eg chemotherapy, radiation therapy) history that may predispose to bone malignancy.
• The presence or absence of systemic features of disease.
• Results of relevant laboratory investigations (see text).

**Table 6:** BONE TUMOUR DIAGNOSIS: RADIOLOGICAL FEATURES

• The precise anatomical location of the lesion in the affected bone (ie epiphyseal, metaphyseal, diaphyseal, medullary, cortical, periosteal or extraosseous in location).
• The size of the lesion
• The matrix composition of the lesion • The nature of the zone of transition or interface between the lesion and surrounding bone. • The pattern of bone destruction
• The presence or absence of infiltration of medullary bone.
• The presence or absence of cortical destruction and soft tissue involvement
• The nature of the periosteal reaction
• The presence of multiple lesions within bone

Relevant clinical information should be provided on the pathology request form and its content should be recorded in the final pathology report. The age of the patient is crucial for bone tumour diagnosis as a number of bone tumours, both benign and malignant, tend to develop most commonly within a given age range. Some tumours and tumour-like lesions have a predilection to arise in certain bones (eg simple bone cyst occurs most often in the proximal humerus of a child or adolescent). Most bone tumours present with bone pain and swelling. Bone pain is dull, aching and characteristically worse at night. Rapid growth is characteristic of some malignant tumours but is also seen in some benign tumours and tumour-like lesions, such as aneurysmal bone cyst, eosinophilic granuloma and osteomyelitis. A history of trauma may be notable in cases where a post-traumatic lesion (eg haematoma) is a possible diagnosis. Local and systemic signs of infection need to be distinguished from those associated with the growth of a bone tumour, such as Ewing's sarcoma. Information regarding a pre-existing skeletal condition should be provided, including developmental conditions where there are multiple skeletal lesions (eg fibrous dysplasia, osteochondromatosis). It is also important to receive information on any relevant extraskeletal disease (eg history of carcinoma) elsewhere in the body. Racial occupational and treatment factors may also be relevant in the assessment of a bone tumour [16,17].

The results of laboratory investigations which may help in evaluating a bone lesion should be communicated to the reporting pathologist [18]. Details of the white blood cell count and erythrocyte sedimentation rate should be noted if there is a possibility that the lesion is a bone infection, eosinophilic granuloma, leukaemia or other haematological malignancy. Ewing's sarcoma and 'toxic' osteoclasts may present with clinical and laboratory features that resemble osteomyelitis. If myeloma is suspected, protein electrophoresis for the identification of monoclonal immunoglobulin components in the serum or urine should be undertaken. Laboratory tests reflecting bone turnover, such as the serum calcium, phosphate and alkaline phosphatase should also be known, particularly if there is a need to exclude a metabolic cause for the development of a bone tumour, such as a "brown tumour" of hyperparathyroidism or Paget's disease. The alkaline phosphatase may also be elevated in osteosarcoma, 'blastic' metastases, fracture, polyostotic fibrous dysplasia and other conditions. The acid phosphatase may be elevated in prostate carcinoma.

Radiological information is essential for bone tumour diagnosis [15,19] and it is strongly recommended that, wherever possible, the pathologist should personally view the radiological images of a bone tumour before issuing a diagnostic report. Where this is not possible, it should be recorded in the pathology report.

The precise anatomical location of a lesion in bone is important because tumours have a tendency not only to develop more commonly in certain bones but also more frequently to involve the particular anatomical region of an affected bone. It should also be evident from the radiology whether a lesion has originated in bone or extended into it from surrounding soft tissues.

The matrix composition of the lesion may point to specific diagnostic possibilities (eg calcification within cartilage tumour or ossification within a bone-forming tumour). The interface between the lesion and surrounding bone, particularly whether the lesion is well or poorly defined, should be noted as this may favour a particular benign or malignant diagnosis. A sclerotic rim is commonly present around slow growing lesions and usually points to a benign diagnosis. A non-sclerotic margin is usually found around a more rapidly growing bone lesion; malignant lesions are commonly poorly defined and have a broad zone of transition.

The pattern of bone destruction should be identified as it indicates the rate of growth of a bone lesion. A geographic pattern of bone destruction is characterised by the presence of well-circumscribed lytic areas (maximum dimension more than 1 cm) with a well-defined margin; this reflects the slow growth rate of these lesions, which are usually benign tumours (eg non-ossifying fibroma) or locally aggressive/low-grade malignant tumours (eg giant cell tumour of bone, low-grade chondrosarcoma). A rim of sclerosis between normal host bone and the lytic area may or may not be present. A moth-eaten pattern of bone destruction is characterised by the presence of multiple small lytic areas (usually 2-5 mm) separated by identifiable bone; this indicates an aggressive pattern of growth and is most often seen in malignant neoplasms, although it can be seen in some forms of osteomyelitis and eosinophilic granuloma. A permeative pattern of bone destruction is characterised by diffuse marrow involvement in which there are multiple tiny lytic areas (< 1 mm maximum dimension). This is usually accompanied by a broad zone of transition and reflects rapid growth of a bone lesion. A permeative pattern occurs in malignant tumours such as Ewing's sarcoma and osteosarcoma, but can also be seen in some benign entities such as osteomyelitis and eosinophilic granuloma.

Radiological evidence of extension of the tumour through the bone cortex and involvement of surrounding soft tissue should be noted as this provides evidence of a locally aggressive or malignant tumour. The nature of the periosteal reaction associated with a bone lesion oftens reflects the growth rate of the tumour. When the tumour grows slowly, the periosteum forms a thick layer of bone. Multiple layers of periosteal new bone are formed when there is a succession of fast and slow growth phases associated with the enlargement of the underlying lesion. The presence of tumour on both sides of the cortex (which is not yet destroyed) often indicates a very aggressive lesion.

The presence of multiple lesions within bone should be determined as this may suggest particular conditions such as multiple cartilage tumours (eg enchondromatosis, multiple osteochondromas) or Langerhans cell histocytosis (LCH). This feature is also useful in assessing whether a malignant tumour is more likely to be primary or secondary. With regard to primary malignant bone tumours, it may also point to a diagnosis of multifocal osteosarcoma or metastatic Ewing's sarcoma.

## 4. General handling of bone tumour specimens before dissection

The following information should be recorded for all bone tumour specimens:-

1. Patient and specimen identification details including patient name, age (date of birth), sex, hospital and surgical pathology identification numbers.

2. Identification of the type of specimen received eg fine or core needle (closed) biopsy, surgical (open) biopsy, curettage, excision (segmental resection/en bloc), limb salvage, amputation or specific type of complex resection (eg hemipelvectomy).

It is often useful to receive specimens fresh (unfixed) in the laboratory. This permits the use of a number of specialised investigations where appropriate. These include the provision of material for frozen section diagnosis, the use of specific fixatives for histochemistry and snap freezing of tissue for molecular genetic studies [1,7,20,21]. Fresh tissue can also be sent for microbiological culture or cytogenetic studies. Where the above studies are not anticipated or where there is likely to be a delay in the processing the specimen should be immediately fixed in 10% neutral buffered formalin. It is important that specimens are not placed in a freezer as this may result in formation of ice crystal artefacts.

It may be useful to specify whether the specimen was received fresh or in fixative and to record details of specimen transport to the laboratory; it should be noted if this has led to a delay or problems in processing. 10% neutral buffered formalin fixation is routinely used in most laboratories and is generally suitable for bone specimens. Fixation in absolute alcohol is useful for identifying glycogen in Ewing sarcoma cells. Small biopsy fragments containing only cancellous bone fragments or tumour tissue containing small amounts of bone can be decalcified and fixed in one overnight step through the use of 5% trichloracetic acid or ethylene diaminetetracetic acid (EDTA) dissolved in 10% buffered formalin.

Decalcification with strong (eg nitric) or weak (eg formic) acid solutions is required for the histological processing of most bone tumour specimens. To shorten the period required for decalcification, samples of a tumour biopsy that are not heavily mineralised should be selected; 2-4 mm thick slices should be submitted for decalcification from large specimens. Where there is a need for rapid diagnosis, 5% nitric or 20% formic acid can be used for rapid decalcification. Immersion of bone biopsy specimens in strong acids should not exceed 24 hours. The mineralisation status of the specimens should be constantly monitored. This is usually carried out by specimen radiography but chemical (eg calcium oxalate) tests are also available to determine the end point of decalcification. EDTA made neutral with sodium hydroxide solution removes calcium more slowly but provides better preservation of tissue and cytological details as well as antigenicity. It should be noted that most ancillary stains can be employed without modification to tissues that are decalcified even in strong acids; the antigenicity of many common epitopes is not abrogated by decalcification in strong acids [22]. However, strong acids can interfere with molecular genetic analysis [23].

In general, decalcified tissue is processed in the same way as undecalcified tissue. After paraffin embedding, blocks should be trimmed, placed in the freezer for 30 minutes and then on an ice tray before 3-5 μm sections are cut. A standard rotary microtome is suitable for routine use; larger sections can be cut on a sledge microtome. Haematoxylin-eosin stains are used routinely for morphological diagnosis. Ehrlich's or Cole's formula for haematoxylin is recommended as it gives the best differential staining of calcified bone, osteoid and cement lines. Other useful ancillary stains include PAS (+/- diastase), reticulin, toluidine blue, Alcian blue, Perls, Congo Red and Masson-Fontana.

## 5. Frozen section and aspiration cytology of bone tumours

Frozen section examination of bone tumours should only be carried out by pathologists who have some experience of osteoarticular pathology and who have knowledge of the clinical background and radiological appearances of the lesion; the usefulness of frozen section histological examination is predicated on close cooperation between the surgeon, radiologist and pathologist [1,2,20,21]. Frozen section histology provides information on:-

• Adequacy of the biopsy specimen.

• The nature of the lesion.

• Ancillary investigations which may be required for diagnosis.

• Adequacy of resection margins.

Frozen section analysis is particularly useful in determining whether the sampled tissue is adequate and representative of the biopsied lesion. It is also used intraoperatively for the examination of resection margins to determine the level of excision or amputation of a bone tumour. Frozen section also has a diagnostic role, often indicating to an experienced pathologist the nature of the lesion. It is particularly useful in this regard in indicating whether a lesion is likely to be inflammatory or neoplastic; this may be helpful in directing the surgeon to take further samples for microbiological culture or carrying out cytogenetic and molecular genetic analysis. In some cases, the appearances of the lesion are sufficiently characteristic to permit a definitive diagnosis; in the appropriate clinical and radiological context, this may permit immediate surgical treatment of the lesion. A definitive diagnosis of bone tumour, however, should not be based on the examination of frozen sections alone. Gross specimens submitted for frozen section should be carefully evaluated for the presence of heavily mineralised tissues, such as fragments of cortical bone, which should be removed from the samples submitted for frozen section. Stained touch or imprint preparations can be used to provide supplementary information to a frozen section diagnosis and are particularly useful if the tissue is heavily mineralised. Imprint preparations are particularly useful in the diagnosis of round cell tumours of bone, such Ewing's sarcoma, lymphoma, and osteosarcoma, but may also occasionally suggest a diagnosis of osteomyelitis, eosinophilic granuloma or carcinoma.

Aspiration cytology can also provide useful information on bone tumours [24-27]. However, it has a relatively limited role in the diagnosis of these neoplasms. As with imprint preparations, when combined with frozen section histology, it can provide important cytological information that may point to a specific diagnosis. It may have a particular role in the diagnosis of possible sarcoma recurrence or metastasis of previously well-documented neoplasms. Under these circumstances, when combined with adequate radiological and clinical information, a diagnosis can often be suggested.

## 6. Bone tumour biopsy and curettage specimen handling

Biopsy specimens for histological examination are usually obtained by either open (surgical) biopsy, or closed (percutaneous) needle biopsy [28-30].

Closed needle biopsy is an effective and safe technique and is often used in the initial diagnosis of a bone tumour. Most lesions can be biopsied in this way, yielding adequate material to permit an experienced pathologist to make a diagnosis. With this technique there is little risk of dissemination of the tumour in the course of the procedure. The principal disadvantage of needle biopsy is that it provides less material for histological examination than an open biopsy; this may limit the amount of diagnostic information that can be obtained from the biopsy. In addition, as much of the biopsy tissue is required for histological diagnosis, this can limit or preclude the use of other investigations ( eg molecular genetics), which may provide supplementary diagnostic information. An open biopsy specimen generally provides more material for histological examination than a closed biopsy. Open biopsy is commonly employed for the diagnosis of bone tumours in children from whom it may be difficult to obtain a closed biopsy specimen. It is recommended that an open biopsy is carried out at the centre where the tumour is to be treated as this ensures that the biopsy tract is removed at the time of definitive surgery. When combined with frozen section examination of the biopsy sample, this technique ensures that the biopsy tissue is adequate and representative of the lesion.

Most biopsy specimens require at least three hours fixation. Core needle biopsy specimens, if properly fixed, maybe decalcified overnight in acid or a chelating agent. If the core is 5 mm or more in thickness, it should be divided. In general, if ancillary studies are anticipated then a minimum of three cores may be needed. A general gross description of the biopsy specimen should be given including dimensions, consistency, colour [5,7,19]. The presence of necrosis, thrombus, fibrin clot, myxoid change, bone cartilage and fibrous tissue should be noted. It may be possible to separate soft areas of the tumour from calcified areas of the tumour; the former may be submitted for frozen section or rapid histological diagnosis Handling of curettage specimens is essentially similar to that for core needle biopsies in terms of gross description and specimen handling. If a large amount of curetted material is received, then this should be sampled extensively (about 1 section per cm. Specimens should be submitted for overnight formalin fixation and decalcification.

The surgical pathology report on a biopsy or curettage specimen should record the morphological and cytological features of the tumour, including degree of pleomorphism, mitotic count and the presence of tumour necrosis. The report should contain the histological diagnosis (or differential diagnosis), indicating where possible details of the specific tumour type (+/- subtype) and tumour grade. Even when a specific diagnosis appears to have been established by means of a biopsy, careful histological study of the resection and amputation specimen needs to be carried out as the same histological features may not be present throughout a tumour and the appearances of the tumour tissue can change over time or as a result of treatment.

## 7. Specimen handling and macroscopic description of an amputation or segmental resection for bone tumour

Orthopaedic amputation and segmental resection specimens are often large and will not fix adequately if left un-dissected in formalin. They should be dealt with promptly after arrival in order to expose the key tissues and tumour for subsequent fixation [5-7,19]. The volume ratio of fixative to specimen large specimens should be at least 3:1.

Before dissecting an amputation or large segmental resection for bone tumour, the pathologist should review the pre-surgical clinical history, relevant radiology and biopsy pathology report(s).

The type of amputation (e.g. left hindquarter amputation, right forequarter amputation, right below nee amputation), or resection (eg left distal femoral resection left second toe amputation) should be noted and a digital photograph of the intact specimen taken from at least two aspects. The following are guidelines for large specimen dissection:

1. Measure and record the length of the amputation or resection specimen in three dimensions. For amputations, the length between the major joints and the distance between the tumour and the osteotomy site and the closest soft tissue amputation site should be noted; for resections, the distance between the palpable tumour and the osteotomy site should be recorded. It may be useful to include a measure of the circumference at the level of the tumour. Specimen radiographs may aid in determining the extent of bone, joint and soft tissue involvement at this stage.

2. Record the size and position of any previous surgical scars. Determine the presence, position, and dimensions of previous biopsy sites in an amputation specimen. For resection specimens, record the size and position of the excised skin ellipse (which should be included in the biopsy tract).

3. Search for the major lymph node groups and identify and place them in separate containers of fixative.

4. Cut a cross section of the proximal bone margin with a bandsaw.

5. Take samples of the major vessels at the amputation site and place in a separate container of fixative.

6. Dissect all the soft tissues (down to the periosteum) around the involved bone. If there is any indication (from the radiographs or at the time of dissection) of soft tissue tumour extension, dissect around this area and keep it in continuity with the bone. Unless the tumour involves an adjacent joint, the entire bone containing the tumour should be excised. If, from the radiographs, the tumour involves an adjacent joint, cut through the adjacent, non-involved bone transversely with the band saw approximately 5-10 cms from the joint, and then excise the involved bone, joint and attached uninvolved length of bone.

7. Once the key part of the amputation specimen has been dissected out, a plain radiograph of the intact tumour and bone can be taken using a laboratory X-ray machine. This will aid the subsequent decision on the preferred initial slice through the specimen.

8. Take tissue samples for histology of any previous surgical sites or biopsy tracts in order to determine the presence/absence of tumour implantation.

9. Long bones containing the tumour (and the excised adjacent joint, if applicable), should be cut longitudinally with a band saw. In most cases, the cut should be in the coronal plane, dividing the specimen into anterior and posterior halves. When the tumour is a surface tumour involving the anterior or posterior aspect of the bone (eg parosteal osteosarcoma, surface chondrosarcoma), a sagittal section may be preferable for tumours of unusual shape/site of origin. It is nearly always sufficient to take an initial coronal slice and then subsequent additional slices to complement the coronal slice. For hindquarter and forequarter amputations, and for pelvic and scapular bone tumours, it may be necessary to cut the bone obliquely; in these bones the ideal cut usually involves the joint (hip and shoulder, respectively) and includes the area of tumour origin/main areas of tumour with subsequent slices used to sample any extra-osseous tumour extension.

10. After fixing the sliced bone specimen for a further 24 hours, a cut is made using a band saw parallel to the initial cut in order to provide a slice of the bone tumour. The thickness of this slice should be approximately 5 mm. This slab should be radiographed to provide a specimen radiograph. For bone resection specimens, the surgical tissue margins should be taken after fixation. Medial, lateral, anterior and posterior (as well as the osteotomy transverse slice) margins should be taken as minimum. Further margins can be taken if there is concern that there is tumour spread; the surgeon should mark with a suture or ink any areas of particular concern alternatively, the surgical margins can be sampled by the pathologist when the surgeon is present. If the tumour has destroyed or breached the cortex, the margin can be sampled with a scalpel or knife.

11. The slab specimen should be photographed.

12. The soft tissues including major vascular structures around the dissected bone of the amputation or resection specimen should be examined. Other apparently uninvolved bones should be sliced sagitally and/or coronally to look for foci of tumour or other lesions and the major joints should be examined.

13. Representative tissue blocks taken for histology from an amputation or resection specimen of a bone tumour should include:-

• Blocks of tumour: a slab of all of the tumour in bone should be blocked out and a photographic record kept of where the tissue blocks for histology have been taken (Figure 1).

**Figure 1:**
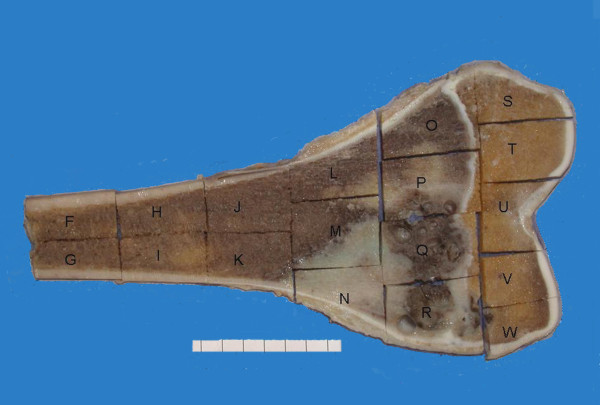
**Photograph of a slab taken through a specimen of an osteosarcoma of the lower femur**. This provides information on the nature and site of sampled tissue blocks, the location of the tumour, its relation to bone resection margins, and permits calculation of the extent of tumour necrosis.

• Additional blocks should be taken from areas of tumour or bone not included in the slab if the macroscopic appearance is unusual.

• Blocks of the previous incision site and biopsy tract (if present).

• Blocks of the proximal bone resection margin at the site of amputation or excision.

• Any abnormal-looking areas elsewhere in bone, soft tissues, or skin.

• Lymph nodes: if grossly normal, only representative ones; if grossly abnormal or if there is clinical suspicion of metastasis, all of them.

• Major vessels at the soft tissue amputation site.

14. The macroscopic description should note the following features:-

• Type and side (left/right) of amputation or bone resection.

• The dimensions of the resection specimen (see above).

• The gross presence or absence of exposed tumour in bone or soft tissue of the specimen.

• The presence, position and dimensions of attached skin and the presence of biopsy sites and surgical scars.

• The anatomical site and location of the tumour within bone (ie whether the lesion is in the epiphysis, metaphysis or diaphysis of a long bone and whether located in the medulla, the cortex or on the surface of the bone).

• Dimensions of the tumour (in centimetres).

• Extent of bone, and soft tissue (including joint) involvement.

• The presence or absence of necrosis (approximate percentage) and other descriptive characteristics (eg colour, calcification, hardness, gritty, haemorrhagic, cystic) should be noted.

• The presence or absence or chemotherapy or radiotherapy effect.

• Involvement or invasion of major structures (eg nerve, major blood vessels).

• Presence of satellite lesions of tumour away from the main tumour mass.

• Presence of lymph nodes and description of cut surface of the nodes.

• Relation of the tumour to the resection margins; the distance (in centimetres) should be measured.

In addition, a record should be kept of whether tissue has been submitted for frozen section, cytogenetics, molecular genetic analysis and other investigations.

## 8. Histological reporting of primary bone tumours

The following histological information should be included in the surgical pathology report:-

• Tumour type (and subtype) according to WHO histological classification.

• Tumour grade (if relevant)

• Morphological and cytological description of the tumour; this may include details of the mitotic count and degree of pleomorphism.

• Tumour necrosis (presence or absence and percentage necrosis in the resection specimen).

• Extent of local tumour spread, in particular whether the tumour involves specific anatomical components or compartments (eg medullary cavity, cortex, joint, extra-osseous soft tissues).

• Status of resection margins with regard to involvement by tumour.

• Results of cytogenetic/molecular genetic investigations (if available).

• SNOMED coding of bone tumour (Table 3).

## 9. Ancillary investigations

### 9.1. Immunohistochemistry

Immunohistochemistry is useful in identifying or confirming the nature of cells in a bone tumour. Immunohistochemistry is particularly valuable in the differential diagnosis of primary and secondary malignant tumours of bone. Expression of epithelial markers, such as epithelial membrane antigen and cytokeratin, may point to a diagnosis of metastatic carcinoma; tumour cell expression of specific antigens (eg PSA, TTF-1) may indicate the likely primary origin. In the paediatric population, expression of NB84a, synaptophysin and chromogranin are useful in the identification of metastatic neuroblastoma [31]. Expression of epithelial markers is also seen in some primary bone tumours, notably adamantinoma and chordoma [32,33]; the former also express podoplanin, and the latter S100 and brachyury [34,35]. Other useful markers include Factor 8 related antigen, CD31, CD34 and podoplanin LYVE-1, which are differentially expressed by endothelial cells in specific vascular tumours [36].

In the diagnosis of malignant round cell tumours of bone, expression of leukocyte common antigen (CD45) indicates that tumour cells are of haematopoietic origin. If a lymphoma is suspected, B and T cell markers, such as CD19/CD20 and CD3 respectively, are useful. Monoclonal kappa or lambda light chain expression can be identified in myeloma or plasmacytoma. Langerhans cells in Langerhans cell histiocytosis can be identified using antibodies directed against S100, and CD1a. Expression of CD99 is useful in confirming the diagnosis of Ewing's sarcoma although it should be appreciated that this antigen can be expressed in other round cell tumours such as in lymphoblastic lymphoma, small cell osteosarcoma and mesenchymal chondrosarcoma [37,38]. Detection of FLI-1, a nuclear protein that is involved in cell proliferation and tumourigenesis, is also useful in the diagnosis of Ewing's sarcoma [39]. FLI-1 is normally expressed by endothelial cells and haematopoietic cells, including T lymphocytes and its expression can be noted in lymphoblastic lymphoma and endothelial cells of vascular neoplasms. WT1, a proliferation marker, which represents a nuclear transcription factor, is also useful in the diagnosis of small round cell tumours, being found in Wilm's tumour, lymphoblastic lymphoma and occasionally neuroblastoma and lymphoma but not Ewing's sarcoma [40]. Mutations in the p53 gene result in the accumulation of p53 protein in the nucleus. p53 expression may be elevated in some malignant tumours of bone and overexpression may be of prognostic significance [41,42].

### 9.2. Cytogenetic analysis

Cytogenetic analysis shows changes in the number and/or structure of chromosomes in a tumour. Some of these chromosomal abnormalities are tumour-specific and are useful in bone tumour diagnosis [43]. Samples of sterile, fresh tumour tissue are required for cytogenetic analysis. A thin slice of non-necrotic tumour tissue should be submitted. The specimen is placed in tissue culture medium (containing serum) and kept at room temperature prior to transport to the laboratory; if there is any delay, the specimen should be kept in a refrigerator (not a freezer). Sample size should be 1 cm^3 ^or more. Cells are grown in short term culture for karyotyping. Chromosome-specific probes can be labelled with fluorescent dyes in order to identify chromosomes or chromosome DNA sequences by fluorescence in situ hybridisation (FISH). Cytogenetic analysis of bone tumours is especially useful in the detection of the 11;22 translocation which is commonly found in Ewing's sarcoma. Cytogenetic abnormalities have also been noted in other tumours including osteochondroma, osteofibrous dysplasia, fibrous dysplasia, giant cell tumour, osteosarcoma, chondrosarcoma and chordoma [3,4,43].

### 9.3 Molecular genetic analysis

Molecular genetic analysis is used to characterise changes in gene and gene expression in tumour cells, particularly translocations and mutations in oncogenes and tumour suppressor genes [3,4]. A variety of methods can be used to identify these genetic abnormalities including reverse transcriptase polymerase reaction (RT-PCR) analysis in which a nucleotide sequence is amplified and then identified by appropriate DNA or RNA probes. Snap frozen tissue is required for optimal molecular genetic analysis using PCR techniques, but both this procedure (and fluorescent *in situ *hybridisation) can be used on paraffin-embedded material. Molecular genetic analysis is particularly useful in the diagnosis of Ewing's sarcoma, which is associated with a characteristic reciprocal translocations that involve the EWS gene on chromosome band 22 q12 and genes from different members of the ETS family of transcription factors [44-46]. The most common of these translocations is t [11; 22] (q24; q12), which is present in nearly 85% of cases of Ewing's sarcoma; this results in a tumourigenic fusion protein composed of the 5'- end of the EWS gene and 3'- end of the ETS family gene FLI1: This EWS-FLI1 fusion product has been reported in other malignant round cell tumours including neuroblastoma and mesenchymal chondrosarcoma [47,48]. The second most common translocation is t(21; 22) (q24, q12), which involves the same segment of the EWS gene combined with the 3'end of the ETS family gene ERG. The EWS gene may combine with other ETS family genes in other translocations including t(7; 22), t(17; 22), and t(2; 22). Molecular studies are also useful in identifying loss or mutation in certain tumour suppressor genes, such as p53 and the retinoblastoma gene, both of which are associated with the pathogenesis of osteosarcoma [49-53]. The loss of both functional alleles by either deletion or mutation results in unrestrained cell growth.

### 9.4. Other ancillary investigations

Flow cytometry is not routinely employed for tumour diagnosis as it does not accurately distinguish between benign and malignant bone tumours of bone. I can, however, provide supportive evidence that may favour the diagnosis of a benign or malignant tumour and in some cases provides information on prognosis and response to therapy [54,55]. Flow cytometry provides a measurement of a number of features including cell size, viability, cell cycle time and DNA ploidy as well as other cytological parameters. With regard to DNA ploidy, tumours are generally divided into diploid and aneuploid. DNA aneuploidy indicates an abnormal amount of DNA in a tumour cell population relative to normal (diploid) cells. DNA aneuploidy has been documented in benign bone tumours and does not equate with malignancy. However, aneuploidy is more prevalent in high grade malignant tumours and is an independent risk factor for predicting metastasis. Electron microscopy is also not employed routinely for tumour diagnosis; it can in some cases help in establishing the diagnosis of some bone tumours [56,57]; for example identifying Birbeck granules in Langerhan's cell histiocytosis or cytoplasmic glycogen in Ewing's sarcoma.

## 10. Assessment of preoperative chemotherapy

Preoperative chemotherapy is commonly used with limb salvage procedures for the treatment of high-grade sarcomas, particularly osteosarcoma and Ewing's sarcoma. To determine the effect of chemotherapy it is generally required to quantify the extent of tumour necrosis as a percentage of the total tumour area [58]. For osteosarcomas, chemotherapy-induced necrosis of 90% or more has a greater than 90% disease-free survival, compared with less than 50% in patients with less than 90% tumour necrosis [58-61]. For Ewing's sarcoma, significant necrosis, according to most series, appears to be roughly defined as greater than 90% of the microscopic tumour mass [62-67]. Tumours demonstrating such massive necrosis are associated with a favourable prognosis, whereas those with less necrosis are associated with poor survival.

To determine the extent of necrosis in an osteosarcoma or Ewing's sarcoma specimen, the slab specimen of the resected bone containing the tumour provides the template for histological analysis [5-7,66]. A photograph or radiograph of the slice is taken and the site of each numbered block marked on a grid pattern diagram. Additional blocks are also taken in a plane at right angles to this set of blocks to determine the full extent of the tumour. As indicated earlier, blocks should also be taken from other representative areas of the specimen including areas of unusual appearance in bone and soft tissue surrounding the tumour and possible satellite lesions in order to get a clear picture of the volume and extent of the tumour. Treated osteosarcomas may contain large atypical cells with hyperchromatic nuclei, smudged or clumped chromatin and vacuolated cytoplasm in areas of necrosis, calcification or fibrosis [66,67]. The nature of these cells is not certain but they are currently considered to represent viable tumour cells in assessing the extent of tumour necrosis. Other effects of chemotherapy on tumour histology include ghost-like cells with loss of nuclear and cytoplasmic detail, granulation tissue formation, fibrosis, haemosiderin deposition, mucinous change and inflammation.

## Competing interests

The authors declare that they have no competing interests.

## Authors' contributions

NAA and DCM both wrote and approved the final manuscript.

## References

[B1] Hogendoorn PC Athanasou NA Bielack S De Alava E Dei Tos AP Ferrari S Gelderblom H Grimer R Hall KS Hassan B Jurgens H Paulussen M Roseman L Taminiau AH Whelan J Vanel D Bone sarcomas: ESMO Clinical Practice Guidelines for diagnosis, treatment and follow-up Ann Oncol 2010 21 Suppl 5 v204 213 20555083 10.1093/annonc/mdq223

[B2] Grimer R Athanasou NA Gerrand C Judson I Lewis I Morland B Peake D Seddon B Whelan J UK Guidelines for the Management of Bone Sarcomas Sarcoma 2010 317462 21253474 10.1155/2010/317462 PMC3022187

[B3] Fletcher CDM Unni KK Mertens F eds Pathology and Genetics of Tumours of Soft Tissue and Bone World Health Organisation Classification of Tumours 2002 Lyon, France: IARC Press

[B4] Unni KK Inwards CY Bridge JA Kindblom LG Wold LE AFIP Atlas of Tumour Pathology; 4^th ^Series, Fascicle 2: Tumours of the Bones and Joints 2005 Washington

[B5] Abdul-Karim FW Bauer TW Kilpatrick SE Raymond KA Siegal GP Recommendations for the reporting of bone tumours Hum Pathol 2004 1173 8 15492983 10.1016/j.humpath.2004.07.004

[B6] Rubin BP Fletcher CD Inwards C Montag AV Peabody T Qualman SJ Rosenberg AE Weiss S Krausz T Protocol for the examination of specimens from patients with soft tissue tumours of intermediate malignant potential, malignant soft tissue tumours, and benign/locally aggressive and malignant bone tumours Arch Pathol Lab Med 2006 130 1616 29 17076523 10.5858/2006-130-1616-PFTEOS

[B7] Rubin BP Antonescu CR Gannon FH Hunt JL Inwards CY Klein MJ Kneisl JS Montag AG Peabody TD Reith JD Rosenberg A Krausz T Members of the Cancer Committee, College of American Pathologists Protocol for the examination of specimens from patients with tumors of bone Arch Pathol Lab Med 2010 134 e1 7 20367293 10.5858/134.4.e1

[B8] Evans HL Ayala AG Romsdahl MM Prognostic factors in chondrosarcoma of bone: a clinicopathologic analysis with emphasis of histologic grading Cancer 1997 40 818 831 890662 10.1002/1097-0142(197708)40:2<818::aid-cncr2820400234>3.0.co;2-b

[B9] Davis AM Bell RS Goodwin PJ Prognostic factors in osteosarcoma. A critical review J Clin Oncol 1994 12 423 431 8113851 10.1200/JCO.1994.12.2.423

[B10] Guillou LJ Coindre JM Bonichon F Nguyen BB Terrier P Colin F Vilain MO Mandard AM Le Doussai V Lerous A Jacquemier J Duplay H Sastre-Garau X Costa J Comparative study of the National Cancer Institute and French Federation of Cancer Centers Sarcoma Group grading systems in a population of 410 adult patients with soft tissue sarcoma J Clin Oncol 1997 15 350 362 8996162 10.1200/JCO.1997.15.1.350

[B11] Enneking WF A system of staging musculoskeletal neoplasms Clin Orthop 1986 204 9 24 3456859

[B12] Sobin LH Gospodarowicz M Wittekind C TNM Classification of Malignant Tumours 2009 7 New York: Wiley-Liss Springer-Verag

[B13] Edge SB Byrd DR Carducci MA Compton CC Fritz AG Greene FL Trotti A AJCC Cancer Staging Manual 2009 7 New York, NY: Springer

[B14] Heck RK Jr Peabody TD Simon MA Staging of primary malignancies of bone CA Cancer J Clin 2006 56 366 375 10.3322/canjclin.56.6.366 17135693

[B15] Vlychou M Athanasou NA Radiological and pathological diagnosis of paediatric bone tumours and tumour-like lesions Pathology 2008 40 196 216 10.1080/00313020701813784 18203042

[B16] Helliwell T T Helliwell Classification and diagnosis of bone neoplasms Pathology of bone and joint neoplasms 1998 WB Saunders 1 14

[B17] Guo W Xu W Huvos AG Healey JH Feng C Comparative frequency of bone sarcomas among different racial groups Clin Med J 1999 112 1101 4 11721448

[B18] Cravern RD Laboratory evaluation of pediatric bone and soft tissue tumors Orthop Clin North Am 1996 27 461 471 8649729

[B19] Ritchie DA Davies AM T. Helliwell Imaging studies in bone neoplasia Pathology of Bone and Joint Neoplasms 1999 Philidelphia WB Saunders 106 128

[B20] Weatherby RP Unni KK Practical aspects of handling orthopaedic specimens in surgical pathology laboratory Pathol Annual 1982 17 1 31 6763671

[B21] Fechner RE Huvos AG Mirra JM Spjut HJ Unni KK A symposium on the pathology of bone tumors Pathol Annual 1984 19 125 194 6087254

[B22] Athanasou NA Quinn J Heryet A Woods CG McGee JO'D Effect of decalcification agents on the immunoreactivity of cellular antigens J Clin Pathol 1987 40 874 878 10.1136/jcp.40.8.874 2443541 PMC1141128

[B23] Mangham DC Williams A McMullan DJ McClure J Sumathi VP Grimer RJ Davies AM Ewing's sarcoma of bone: the detection of specific transcripts in a large, consecutive series of formalin-fixed, decalcified, paraffin-embedded tissue samples using the reverse transcriptase-polymerase chain reaction Histopathology 2006 48 363 376 10.1111/j.1365-2559.2006.02318.x 16487358

[B24] Layfield LJ Dodd LG T Helliwell Fine needle aspiration of bone and joint neoplasms Pathology of bone and joint neoplasms 1999 Philedelphia WB Saunders 129 156

[B25] Bommer KK Ramzy I Mody D Fine-needle aspiration biopsy in the diagnosis and management of bone lesions: a study of 450 cases Cancer 1997 81 148 56 10.1002/(SICI)1097-0142(19970625)81:3<148::AID-CNCR4>3.0.CO;2-N 9196013

[B26] Willen H Fine needle aspiration in the diagnosis of bone tumours Acta Orthop Scand (Suppl) 1997 273 47 53 9057587 10.1080/17453674.1997.11744702

[B27] Kilpatrick SE Ward WG Chauvenet AR Gold SH The role of fine-needle aspiration biopsy in the intial diagnosis of pediatric bone and soft tissue tumours:An institutional experience Mod Pathol 1998 11 923 928 9796716

[B28] Stoker DJ Cobb JP Pringle JA Needle biopsy of musculoskeletal lesions. A review of 208 procedures J BoneJoint Surg 1991 73B 498 500 10.1302/0301-620X.73B3.1670457 1670457

[B29] Welker JA Henshaw RM Jelinek J Schmookler BM Malawer MM The percutaneous needle biopsy is safe and recommended in the diagnosis of musculoskeletal masses. Outcomes analysis of 155 patients at a sarcoma referral centre Cancer 2000 89 2677 81 10.1002/1097-0142(20001215)89:12<2677::AID-CNCR22>3.0.CO;2-L 11135231

[B30] Kilpatrick SE Cappellari JO Bos GD Gold SH Ward WG Is fine-needle aspiration biopsy a practical alternative to open biopsy for the primary diagnosis of sarcoma? Experience with 140 patients Am J Clin Pathol 2001 115 59 68 10.1309/YN14-K8U4-5FLJ-DGJE 11190808

[B31] Miettinen M Chatten J Paetau A Stevenson A Monoclonal antibody NB84a in the differential diagnosis of neuroblastoma and other small round cell tumors Am J Surg Pathol 1998 22 327 32 10.1097/00000478-199803000-00007 9500774

[B32] Benassi MS Campanacci L Gamberi G Ferrari C Picci P Sangiorgi L Campanacci M Cytokeratin expression and distribution in adamantinoma of the long bones and osteofibrous dysplasia of tibia and fibula. An immunohistochemical study correlated to histogenesis Histopathology 1994 25 71 6 10.1111/j.1365-2559.1994.tb00600.x 7525449

[B33] Meis J Giraldo AA Chordoma: an immunohistochemical study of 20 cases Arch Pathol Lab Med 1988 102 553 556 2451900

[B34] Kashima TG Dhonge A Taylor R Flanagan AM Hogendoorn P Athanasou NA Podoplanin expression in adamantinoma of long bones and osteofibrous dysplasia Virchows Archiv 2011 in press 10.1007/s00428-011-1081-2 21499851

[B35] Vujovic S Henderson S Presneau N Odell E Jacques TS Tirabosco R Boshoff C Flanagan AM Brachyury, a crucial regulator of notochordal development, is a novel biomarker for chordomas J Pathol 2006 209 157 65 10.1002/path.1969 16538613

[B36] Hu X Edwards J de Silvio O Jackson D Banerji S Athanasou NA Expression of a lymphatic endothelial cell marker in benign and malignant vascular tumours Human Pathology 2004 35 857 861 10.1016/j.humpath.2004.02.009 15257549

[B37] Riopel M Dickman PS Link MP Perlman EJ MIC2 analysis in pediatric lymphomas and leukemias Hum Pathol 1994 25 396 9 10.1016/0046-8177(94)90149-X 8163272

[B38] Folpe AL Hill CE Parham DM O'Shea PA Weiss SW Immunohistochemical detection of FLI-1 protein expression: A study of 132 round cell tumours with emphasis of CD99-positive mimics of Ewings's sarcoma/primitive neuroectodermal tumour Am J Surg Pathol 2004 24 1657 62 11117787 10.1097/00000478-200012000-00010

[B39] Folpe AL Chand EM Goldblum JR Weiss SW Expression of FLI-1, a nuclear transcription factor distinguishes vascular neoplasms from potential mimics Am J Surg Pathol 2001 25 1061 6 10.1097/00000478-200108000-00011 11474291

[B40] Hill DA Pfeifer JD Marley EF Dehner LP Humphrey PA Zhu X Swanson PE WT1 staining reliably differentiates desmosplastic small round cell tumour from Ewing sarcoma/primitive neuroectodermal tumour Am J Clin Pathol 2000 114 345 53 10989634 10.1093/ajcp/114.3.345

[B41] Hung J Anderson R p53: function, mutations and sarcomas Acta Orthop Scand 1997 68 Suppl 273 68 73 10.1080/17453674.1997.11744705 9057590

[B42] Lonardo F Ueda T Huvos AG Ladaryi M p53 and MDM2 alterations in osteosaromas: Correlation with clinicopathologic features and proliferative rate Cancer 1997 79 1541 7 10.1002/(SICI)1097-0142(19970415)79:8<1541::AID-CNCR15>3.0.CO;2-Y 9118036

[B43] Bridge JA Orndal CC T. Helliwell Cytogenetic analysis of bone and joint neoplasms Pathology of bone and joint neoplasms 1998 Philadelphia. WB Saunders 59 78

[B44] Ladanyi M The emerging molecular genetics of sarcoma translocations Diagn Mol Pathol 1995 4 214 9 10.1097/00019606-199509000-00003 7493135

[B45] May WA Lessnick SL Braun BS Klemsz M Lewis BC Lunsford LB Hromas R Denny CT The Ewing's sarcoma EWS/FLI-1 fusion gene encodes a more potent transcription activator and is a more powerful transforming gene than FLI-1 Mol Cell Biol 1993 13 7393 8 8246959 10.1128/mcb.13.12.7393-7398.1993 PMC364810

[B46] Zielenska M Zhang ZM Ng K Marrano P Bayani J Ramierez OC Sorensen P Thorner P Greenberg M Squire JA Acquisition of secondary structural chromosomal changes in paediatric Ewing sarcoma is a probable prognostic factor for tumour response and clinical outcome Cancer 2001 91 2156 64 10.1002/1097-0142(20010601)91:11<2156::AID-CNCR1244>3.0.CO;2-I 11391597

[B47] Thorner P Squire J Chilton-McNeill S Marrano P Bayani J Malkin D Greenberg M Lorfenzana A Zielenska M Is the EWS/FLI-1 fusion transcript specific for Ewing's sarcoma and peripheral primitive neuroectodermal tumor? A report of four cases showing this transcript in a wider range of tumour types Am J Pathol 1996 148 1125 38 8644855 PMC1861517

[B48] Burchill SA Wheeldon J Cullinane C Lewis IS EWS-FLI-1 fusion transcripts identified in patients with typical neuroblastoma Eur J Cancer 1997 33 239 43 10.1016/S0959-8049(96)00463-7 9135495

[B49] Sainati L Scapinello A Montaldi A Bolcato S Ninfo V Carli M Basso G A mesenchymal chondrosarcoma of a child with a reciprocal tanslocation (11;22)(q24;q12) Cancer Genet Cytogenet 1993 71 144 7 10.1016/0165-4608(93)90020-M 8281518

[B50] Araki N Uchida A Kimura T Yoshikawa H Aoki Y Uedea T Takai S Miki T Orio K Involvement of the retinoblastoma gene in primary osteosarcoma and other bone and soft-tissue tumors Clin Orthop 1991 270 271 7 1884549

[B51] Ueda Y Dockhorn-Dworniczak B Blasius S Mellin W Wuisman P Bocker W Roessner A Analysis of mutant p53 protein in osteosarcomas and other malignant and benign lesions of bone J Cancer Res Clin Oncol 1993 119 172 8 10.1007/BF01229533 8418091 PMC12201587

[B52] Feugeas O Guriec N Babin-Boilletot A Marcellin L Simon P Babin S Thyss A Hofman P Terrier P Kalita C Brunat-Mentigny M Patricot LM Oberling F Loss of heterozygosity of the Rb gene is a poor prognostic factor in patients with osteosarcoma J Clin Oncol 1996 14 467 72 8636759 10.1200/JCO.1996.14.2.467

[B53] Miller CW Aslo A Won A Tan M Lampkin B Koeffler HP Alterations of the p53, Rb and MDM2 genes in osteosarcoma J Cancer Res Clin Oncol 1996 122 559 65 10.1007/BF01213553 8781571 PMC12200418

[B54] Xiang JH Spanier SS Benson NA Braylan RC Flow cytometric analysis of DNA in bone and soft-tissue tumors using nuclear suspensions Cancer 1987 59 1951 1958 10.1002/1097-0142(19870601)59:11<1951::AID-CNCR2820591119>3.0.CO;2-X 3032396

[B55] Mandahl N Baldetorp B Ferno M Akerman M Rydolm A Heim S Willen H Killander D Mitelman F Comparative cytogenetic and DNA flow cytometric analysis of 150 bone and soft-tissue tumors Int J Cancer 1993 53 358 364 10.1002/ijc.2910530303 8428789

[B56] Hirohata K Morimoto K Ultrastructure of bone and joint diseases Excerpta Medica 1971

[B57] Roessner A Grundmann E Electron microscopy in bone tumor diagnosis Curr Top Pathol 1982 71 153 199 7116948 10.1007/978-3-642-68382-4_6

[B58] Picci P Bacci G Campanacci M Gasparini M Pilotti S Cerasoli S Bertoni F Guerra A Capanna R Albisinni U Histologic evaluation of necrosis in osteosarcoma induced by chemotherapy. Regional mapping of viable and nonviable tumour Cancer 1985 56 1515 21 10.1002/1097-0142(19851001)56:7<1515::AID-CNCR2820560707>3.0.CO;2-6 3861228

[B59] Raymond AK Chawla SP Carrasco CH Ayala AG Fanning CV Grice B Armen T Plager C Papadopoulos NE Edeiken J Osteosarcoma chemotherapy effect: A prognostic factor Semin Diagn Pathol 1987 4 212 236 3313606

[B60] Coffin CM Lowichik A Zhou H Treatment effects in pediatric soft tissue and bone tumours: practical considerations for the pathologist Am J Clin Pathol 2055 123 75 90 15762282 10.1309/h0d4vd760nh6n1r6

[B61] Picci P Sangiorgi L Rougraff BT Nell JR Casadei R Campanacci M Relationship of chemotherapy-induced necrosis and surgical margins to local re-currence in osteosarcoma J Clin Oncol 1994 12 2699 2705 7989947 10.1200/JCO.1994.12.12.2699

[B62] Picci P Rougraff BT Bacci G Neff JR Sangiorgi L Cazzola A Baldini N Ferrari S Mercuri M Ruggieri P Prognostic significance of histopathologic response to chemotherapy in nonmetastatic Ewing's sarcoma of the extremeities J Clin Oncol 1993 11 1763 9 8355043 10.1200/JCO.1993.11.9.1763

[B63] Van der Woude HJ Bloem JL Taminiau AHM Nooy MA Hogendoorn PC Classification of histopathologic changes following chemotherapy in Ewing's sarcoma of bone Skeletal Radiol 1994 23 501 7 7824975 10.1007/BF00223077

[B64] Van der Woude HJ Bloem JL Holscher HC Nooy AH Hermans J Falke TH Hogendoorn PC Monitoring the effect of chemotherapy in Ewing's sarcoma of bone with MR imaging Skeletal Radiol 1994 23 493 500 7824974 10.1007/BF00223076

[B65] Picci P Bohling T Bacci G Ferrari S Sangiorgi L Mercuri M Ruggieri P Manfrini M Ferraro A Casadei R Benassi MS Mancini AF Rosito P Cazzola A Barbieri E Tienghi A Brach del Prever A Comandone A Bacchini P Bertoni F Chemotherapy-induced tumour necrosis as a prognostic factor in localized Ewing's sarcoma of the extremities J Clin Oncol 1997 15 1553 1559 9193352 10.1200/JCO.1997.15.4.1553

[B66] Bacci G Ferrari S Bertoni F Rimondini S Longhi A Bacchini P Forni C Manfrini M Donati D Picci P Prognostic factors in nonmetastatic Ewing's sarcoma of bone treated with adjuvant chemotherapy: analysis of 359 patients at the Instituto Ortopedico Rizzoli J Clin Oncol 2000 18 4 11 10623687 10.1200/JCO.2000.18.1.4

[B67] Wunder JS Paulian G Huvos AG The biological response to chemotherapy as a predictor of the oncological outcome of operative treatment of Ewing sarcoma J Bone Joint Surg Am 1998 80 1020 1033 10.1302/0301-620X.80B6.9012 9698007

